# Ghrelin enhances cisplatin sensitivity in HO-8910 PM human ovarian cancer cells

**DOI:** 10.1186/s13048-021-00907-9

**Published:** 2021-11-17

**Authors:** Yun Leng, Can Zhao, Guoliang Yan, Shuangyue Xu, Yinggui Yang, Ting Gong, Xin Li, Chenglin Li

**Affiliations:** 1 Shenzhen Key Laboratory of Viral Oncology, The Clinical Innovation & Research Center (CIRC), Shenzhen Hospital, Southern Medical University, Shenzhen, 518101 China; 2 School of Pharmaceutical Sciences (Shenzhen), Sun Yat-sen University, Guangzhou, 518101 China; 3 School of Medicine, Xiamen University, Xiamen, 361100 China

**Keywords:** Ghrelin, Cisplatin, Ovarian cancer, Cell cycle, S phase arrest, CDK1

## Abstract

**Background:**

Resistance to platinum-based chemotherapy is one of the crucial problems in ovarian cancer treatment. Ghrelin, a widely distributed peptide hormone, participates in a series of cancer progression. The aim of this study is to determine whether ghrelin influences the sensitivity of ovarian cancer to cisplatin, and to demonstrate the underlying mechanism.

**Methods:**

The anti-tumor effects of ghrelin and cisplatin were evaluated with human ovarian cancer cells HO-8910 PM in vitro or in vivo. Cell apoptosis and cell cycle were analyzed via flow cytometry assay. The signaling pathway and the expression of cell cycle protein were analyzed with Western Blot.

**Results:**

Our results showed that treatment with ghrelin specifically inhibited cell proliferation of HO-8910 PM and sensitized these cells to cisplatin via S phase cell cycle arrest, and enhanced the inhibitory effect of cisplatin on tumor growth of HO-8910 PM derived xenografts in vivo. Treatment with ghrelin inhibited the expression of p-Erk1/2 and p-p38, which was opposite the effect of cisplatin. However, under the treatment of ghrelin, cisplatin treatment exhibited a stronger effect on inhibiting P21 expression, upregulating p-CDK1 and cyclin B1 expression, and blocking cell cycle progression. Mechanistically, ghrelin promoted S phase cell cycle arrest and upregulated p-CDK1 and cyclin B1 expression induced by cisplatin via inhibition of p38.

**Conclusion:**

This study revealed a specifically inhibitory effect of ghrelin on platinum-resistance via suppressing p-P38 and subsequently promoting p-CDK1 mediated cell cycle arrest in HO-8910 PM.

## Introduction

Ovarian cancer is one of the most common causes of mortality among all cancers in females and is the leading cause of mortality from gynecological malignancies, with a poor 5-year survival rate of 38.9% in China [1]. Reasons for this high mortality rate include diagnosis at advanced stages and acquired resistance to chemotherapeutic drugs such as cisplatin and carboplatin [2]. For example, high-grade serous cancers are initially extremely sensitive to platinum-based chemotherapy, with response rates to chemotherapy and surgery being as high as 85%. Paradoxically, though, outcomes are poor, with 5-year survival being less than 30% and relapses typically being characterized by the progressive development of drug resistance [3]. Indeed, almost 80% of the women diagnosed with advanced stages of HGSOC experience recurrence as a drug resistant cell population in tumors that ultimately results in fatality [4].

Platinum-based chemotherapy is the recommended first-line chemotherapy for ovarian cancer treatment. Development of resistance to platinum-based chemotherapy is one of the crucial problems in ovarian cancer treatment. Patients experiencing relapses later than 6 months following a response to platinum-based therapy are characterized as having platinum-sensitive disease, whereas patients who experience recurrences within 6 months following an initial response to platinum-based therapy are defined as having platinum-resistant ovarian cancers [5]. Therefore, reducing platinum-resistant is an important way to suppress relapses and the mortality rate.

Ghrelin is a 28 amino acid peptide which was first identified as a ligand of growth hormone (GH) secretagogue receptors (GHSRs) that regulates GH secretion in 1999 [6]. It plays a critical role in energy balance, food intake and weight regulation and reproduction [7]. Ghrelin prevented mechanical hyperalgesia and cachexia [8, 9], muscle wasting [10], renal dysfunction [11] and testicular damage [12] in patients receiving cisplatin-based chemotherapy; in turn, treatment with cisplatin inhibits ghrelin expression [13]. Moreover, ghrelin promotes a series of cancer progression including cell proliferation [14, 15], cell migration and invasion [16], and angiogenesis. However, whether ghrelin regulates sensitivity of cancer cells to cisplatin chemotherapy is unknown.

Ghrelin and its receptor were expressed in the female reproductive system [17, 18], including oocytes as well as somatic follicular cells, luteal cells from young, mature, old, and regressing corpus luteum (CL), interstitial hilus cells, and ovarian surface epithelium. The Markowska group reported an elevated blood concentration of ghrelin in patients with benign ovarian tumors and ovarian cancer as compared to the control group [19], implying that ghrelin is associated with ovarian cancer.

Ghrelin is found to exist as two major forms in circulation: acyl-ghrelin (AG) and unacylated-ghrelin (UAG). In this article, we studied the effects of acyl-ghrelin on the phenotypes and the chemosensitivity of ovarian cancer to cisplatin treatment and illustrated the underlying mechanism.

## Materials and methods

### Cell lines and regents

The HO-8910 PM human ovarian epithelial carcinoma cell line was purchased from Nanjing KeyGen Biotech Co., Ltd. (Nanjing, China) and cultured in RPMI-1640 medium (Hyclone, Logan, UT, USA) supplemented with 10% FBS. The A2780 human ovarian epithelial carcinoma cell line and its cisplatin resistant cell line A2780CP were purchased from ECACC, UK, and cultivated in RPMI-1640 medium containing 10% FCS and 1.5% L-glutamine. Human induced pluripotent stem cell derived mesenchymal stem cells (iPSC-MSC) and human umbilical vein endothelial cells (HUVEC) were cultured as previously described [20]. The GIST-T1 human gastrointestinal stromal tumor cell line was purchased from Cosmo Bio (Tokyo, Japan). All of the cell lines were kept in a humidified chamber with 5% CO2 at 37 °C. Recombinant human acyl-ghrelin was purchased from AnaSpec (San Jose, CA, USA). Cisplatin (cis-diamminedichlorplatinum, DDP) was purchased from Sigma (Sigma Aldrich, St. Louis, USA). The signaling inhibitors, U0126, for Erk1/2, and SB202190, for p38, were purchased from CST (Cell Signal Technology, Beverly, MA).

### MTT assay

Cells (1–2 × 10^3^) were seeded in 96-well plates and incubated for 24 h for stabilization. Cells were then treated with ghrelin, cisplatin, U0126 or SB202190 for indicated time durations. When groups were treated with U0126 or SB202190, equal amounts of DMSO were supplied in other groups. Next, 5 mg·mL^− 1^ MTT solution (3-(4,5-dimethylthiazol-2-yl)-2,5-diphenyltetrazolium bromide) was added to each well, followed by 3 h’ incubation at 37 °C. The absorbance of the resulting formazan crystals dissolved in DMSO (200 μL) was measured at 570 nm (reference wavelength at 690 nm) using a microplate reader (Model 680 reader; Bio-Rad Laboratories, Hercules, CA).

### BrdU incorporation assay

The BrdU immunoassay (Roche Diagnostics, Indianapolis, IN) was used to determine cell proliferation based on BrdU incorporation during DNA synthesis. In brief, cells were plated on 96-well plates and treated with ghrelin (10 nM) along or with cisplatin for 12 h after cell stabilization. BrdU was then added and the cells were re-incubated for another 12 h. After removing the culture medium, the cultures were then fixed, blocked and incubated with anti-BrdU antibody. The optical density values were measured at 450 nm using the microplate reader (Model 680 reader; Bio-Rad Laboratories, Hercules, CA).

### Cell clone formation assay

Cells were seeded in 6-well plates (500 cells/well) in RPMI-1640 containing 10% FBS with or without ghrelin (10 nM) for 7 days. Medium containing ghrelin was changed every 2 days. Then, cells were fixed by 4% paraformaldehyde after removing the culture medium and stained with crystal violet. The OD (optical density) value was qualified after extraction using 10% acetic acid at 590 nm.

### Cell apoptosis assay

Cell apoptosis was detected using the Annexin V-FITC/Propidium Iodide (PI) kit (Miltenyi Biotec, Bergisch Gald-bach, Germany). In brief, 2 × 10^5^ cells/well were seeded into 6-well plates for stabilization and cultured inwith RPMI-1640 containing 2% (V/V) FBS with indicated administration (10 nM ghrelin, or ghrelin with 1 μg / ml cisplatin, or 5 μM U0126, or 5 μM SB202190) for 48 h. Cells were then dissociated with 0.25% trypsin solution without EDTA (Hyclone, Logan, UT) and stained with 10 μl of Annexin V-FITC for 15 min in the dark at room temperature. After washing, the cells were stained with 5 μl of PI solution immediately prior to analysis by flow cytometer (Beckman-Coulter, Miami, FL).

### Cell cycle analysis

Cells (2 × 10^5^/well) were cultured in 6-well plates in completed medium for stabilization and then cultured with RMPI-1640 containing 2%(V/V)FBS for 24 h. Subsequently, cells were treated with indicated administration (10 nM ghrelin, or ghrelin with 1 μg / ml cisplatin or 5 μM U0126 or 5 μM SB202190) for 48 h. The cells were then trypsinized, fixed and permeabilized with 70% ethanol for overnight. The cells were then washed with PBS and labeled with DAPI (4,6-diamidino-2-phenylindole) staining buffer (Partec GmbH, Münster, Germany). Cell cycle assays were performed with a Partec flow cytometer (Partec GmbH) and analyzed with Modfit LT 5.0 software (Verity Software House, Topsham, ME).

### Immunoblotting

Cell lysates were prepared by using RIPA buffer with protease inhibitor (Pierce, Rockford, IL). Equal amounts of protein (30 μg per lane) were separated by SDS-PAGE and transferred to polyvinylidene difluoride membranes (Roche Diagnostics, Indianapolis, IN). The membrane was blotted with 10% nonfat milk, washed and then probed with antibodies to phosphorylation of p38 (1:2000), phosphorylation of ERK1/2 (1:2000), phosphorylation of CDK1 at Tyr15 and Thr14 (1:500), cyclin B1, P21 (1:1000) and actin (1:1000). All the primary and secondary antibodies were purchased from CST. After washing, the membrane was incubated with horseradish peroxidase-conjugated anti-mouse or anti-rabbit antibody and then visualized by Enhanced Chemiluminescence Plus (Millipore Corporation, Billerica, MA, USA) according to the manufacturer’s protocol.

### Animal studies

6–8-week-old female BALB/c nude mice (Shanghai SLAC Laboratory Animal Co. Ltd., Shanghai, China) were divided into four groups: control, AG, DDP and AG + DDP, and were subcutaneously injected with HO-8910 PM ovarian cancer cells (1 × 10^7^) into the right flanks. One-week post-injection (day 7), the length (a) and width (b) of the tumor were monitored using calipers and the mice were weighted. Then the mice were intraperitoneally injected with AG, DDP, or AG + DDP according to the group set. The measurement of tumor volume and body weight and the injection of drugs were performed every 4 days. The dose of AG and DDP were set as 1 nM/ g and 1 μg/ g according to the body weight of mice. The control group were injected with an equal volume of PBS. The tumor volume (V) was calculated as follows: V = πab^2^/6. At the end of the experiment (day 23), the mice were sacrificed and the xenograft tumors were measured.

### Statistical analysis

All animal data were represented as the means ± SEM, and the other data were represented as the means ± SD. Data analysis was performed through the application of SPSS software for Windows (SPSS, Chicago, Ill). Significant differences between groups were analyzed by unpaired Student’s t-test. One-way ANOVA was followed by the LSD test. A value of *p* < 0.05 was considered to be statistically significant.

## Results

### Ghrelin inhibited cell growth of HO-8910 PM by regulating cell cycle progress

**Fig. 1:**
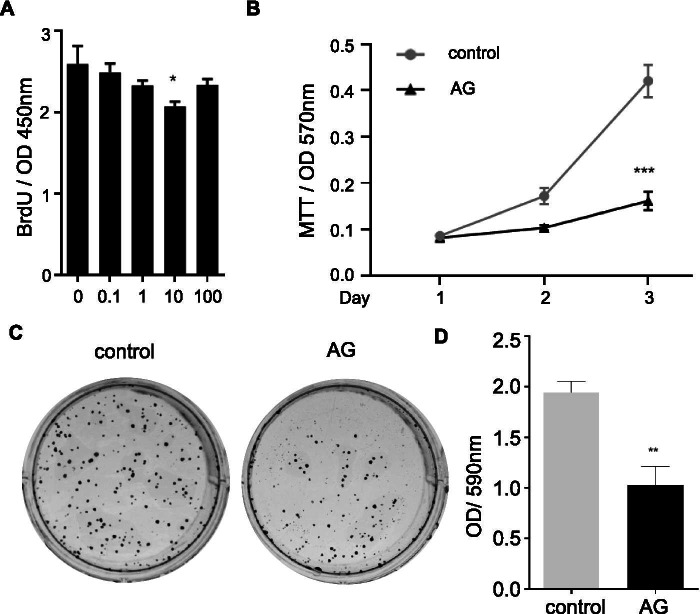
Ghrelin inhibited HO-8910 PM cell growth. **A** BrdU incorporation by HO-8910 PM treated with ghrelin at 0, 0.1, 1, 10, and 100 nM. **B** MTT assay of HO-8910 PM under 10 nM ghrelin treatment at indicated times. **C-D** Plate colony formation assay shows the images of HO-8910 PM treated with 10 nM ghrelin for 7 days (**C**) and the quantification of plate colony formation assay (**D**). Data are shown as mean ± SD from three independent experiments. * *p* < 0.05, **p < 0.01

**Fig. 2:**
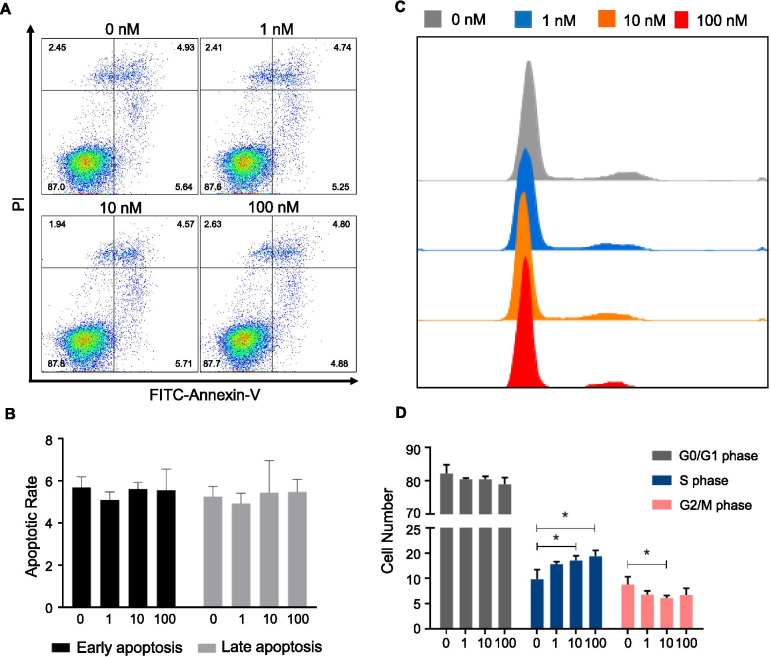
The effects of ghrelin on cell apoptosis and HO-8910 PM cell cycle. **A-D** Representative apoptosis (**A**-**B**) and cell cycle (**C**-**D**) assay of HO-8910 PM treated with different concentrations of ghrelin (0, 1, 10, and 100 nM) for 48 h. Images are representative of three separate experiments. Data are shown as the mean ± SD from three independent experiments. *p < 0.05

BrdU incorporation assay was used to test the effects of ghrelin on cell proliferation of HO-8910 PM. Ghrelin treatment inhibited BrdU incorporation in HO-8910 PM in a dose-dependent manner and showed an optimal inhibitory effect at the concentration of 10 nM (Fig. 1A). Thus, ghrelin was used at 10 nM for the later experiments. Ghrelin also showed an inhibitory effect on cell viability (Fig. 1B) and clone formation of HO-8910 PM (Fig. 1C-D). To understand how ghrelin regulated cell growth, cell apoptosis and cell cycle were tested. In the cell apoptosis assay, there was no distinction among groups with different doses of ghrelin (Fig. 2 A-B). Cell cycle assay demonstrated that ghrelin treatments did not affect the cell cycle in the G0/G1 phase. Nevertheless, increased cells were arrested in S phase under ghrelin treatment, while the percentage of cells in G2/M phase declined, especially in the group treated with ghrelin at 10 nM (Fig. 2C-D). These data suggested that ghrelin inhibited cell growth via cell cycle arrest in HO-8910 PM human ovarian cancer cells.

### Treatment of ghrelin sensitizes HO-8910 PM to cisplatin in vitro

**Fig. 3:**
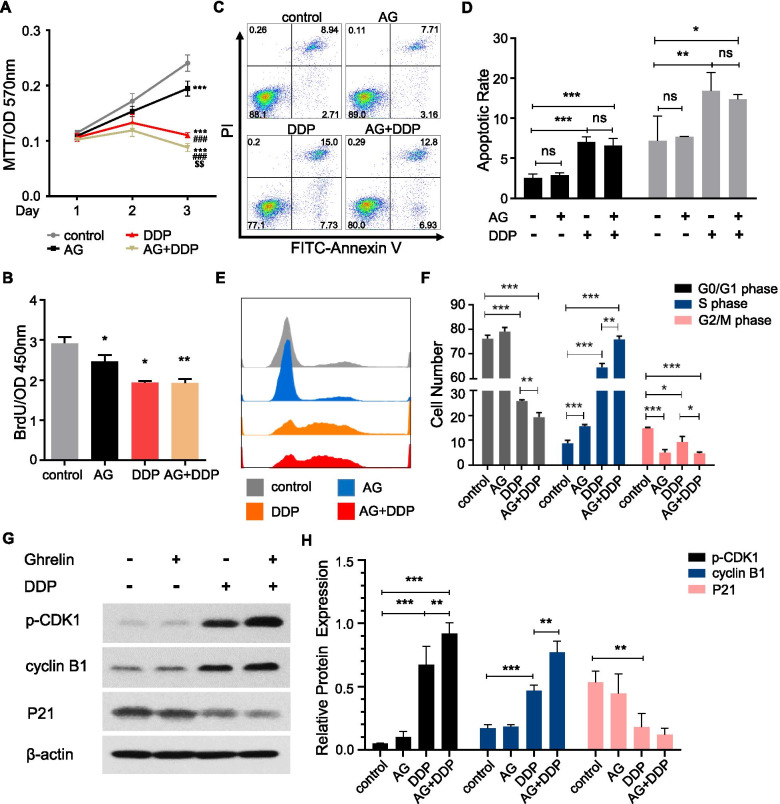
Ghrelin enhanced the suppression of tumor growth by cisplatin in vitro. **A-B** Representative of MTT assay (**A**) or BrdU incorporation assay (**B**) of HO-8910 PM under ghrelin (10 nM) treatment in the presence or absence of cisplatin (1 μg/ml). **C**-**F** Alterations of the apoptosis (**C-D**) and the cell cycle distribution (**E-F**) measured in HO-8910 PM after treatment with ghrelin, cisplatin and the combination of ghrelin with cisplatin. **G-H** Western blotting analysis of the expression of p-CDK1, cyclin B1, and p21 in HO-8910 PM after treatment with ghrelin, cisplatin and the combination of ghrelin with cisplatin for 12 h. β-actin was used as a loading control. Data are shown as the mean ± SD from three independent experiments. *p < 0.05, **p < 0.01, ***p < 0.001

Next, we evaluated whether ghrelin affects tumor suppression by cisplatin in HO-8910 PM. Compared with the control group, both ghrelin and cisplatin treatment inhibited cell viability (Fig. 3A), as well as cell BrdU incorporation of HO-8910 PM (Fig. 3B). However, ghrelin enhanced the suppressive effect of cisplatin on cell viability but not on cell proliferation in HO-8910 PM. Cisplatin exhibited a significant effect in cell apoptosis induction, while ghrelin treatments did not influence cell apoptosis with or without cisplatin (Fig. 3C-D). Moreover, we investigated the effects of ghrelin and cisplatin on the cell cycle of HO-8910 PM. We found that cisplatin treatments inhibited cell populations in G0/G1 phase and G2/M phase but elevated those in S phase (Fig. 3E-F). Ghrelin treatment led to an increased percentage of cells in S phase, but a decreased percentage of cells in G2/M phase (Fig. 3E-F). The combination of cisplatin and ghrelin enhanced the regulatory effect on G0/G1 and G2/M phase inhibition and on S phase arrest (Fig. 3E-F). We subsequently investigated the effects of ghrelin and cisplatin on the protein expression participating in cell cycle progression. We found that cisplatin treatment significantly increased cyclin B1 and phosphorylation of CDK1 (p-CDK1) expression, but decreased p21 expression (Fig. 3G-H). Ghrelin treatment did not alter p-CDK1, cyclin B1 or P21 expression; however, compared with cisplatin treatment, the combination of cisplatin and ghrelin led to further increased expression of p-CDK1 and cyclin B1, and a further decrease in expression of P21 (Fig. 3G-H). These data determined that ghrelin enhanced cisplatin induced tumor inhibition via cell cycle regulation.

### Ghrelin enhanced the suppression by cisplatin on tumor growth in vivo

**Fig. 4:**
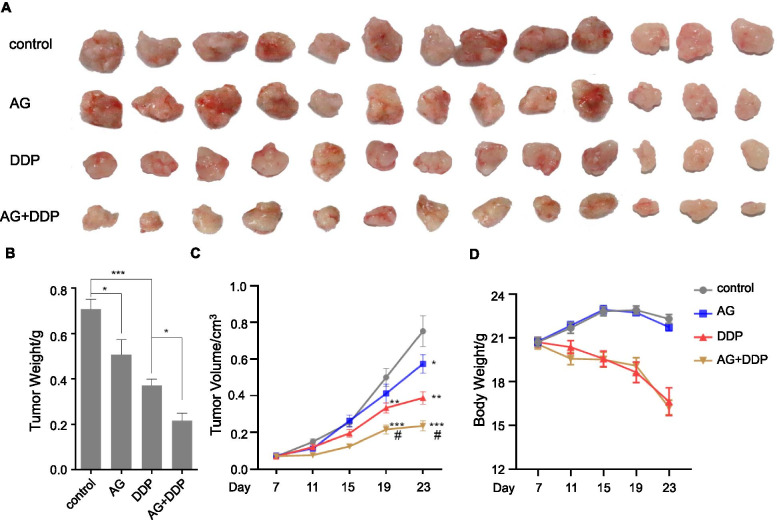
Ghrelin enhanced the suppression of cisplatin on tumor growth in vivo. Mice were subcutaneously injected with HO-8910 PM ovarian cancer cells. One week after injection, mice were treated with PBS (control group), ghrelin, cisplatin and the combination of ghrelin with cisplatin every 4 days. **A** Images of the excised tumors from nude mice. *n* = 13 each group. **B** Average growth curves of tumors in nude mice. **C** Tumor weights of the excised tumors. **D** Body weight curves of the recipient mice. The data are presented as mean ± SEM. * *p* < 0.05, **p < 0.01, ***p < 0.001versus control group. #*p* < 0.01 versus DDP group

To determine the combinatorial effect of ghrelin and cisplatin on tumor formation in vivo, HO-8910 PM cells were subcutaneously injected into female nude mice, followed by ghrelin or cisplatin treatment. We observed that, compared with the control group, ghrelin treatment slightly inhibited tumor volume, but cisplatin treatment inhibited it significantly (Fig. 4A-B). Compared to the group treated with cisplatin, combination with ghrelin enhanced the inhibitory effect of cisplatin on tumor volume to a greater extent than treatment with cisplatin alone (Fig. 4A-B). The tumor weight measured was consistent with the tumor volume (Fig. 4C). We also determined the body weights of recipient mice treated with ghrelin and cisplatin. Cisplatin treatment, but not ghrelin, led to decreased body weight of recipient mice versus the control group (Fig. 4D). The combination of ghrelin with cisplatin exhibited a similar effect as the cisplatin group on the body weight (Fig. 4D).

### Signaling pathways involved in the treatment with ghrelin and cisplatin

**Fig. 5:**
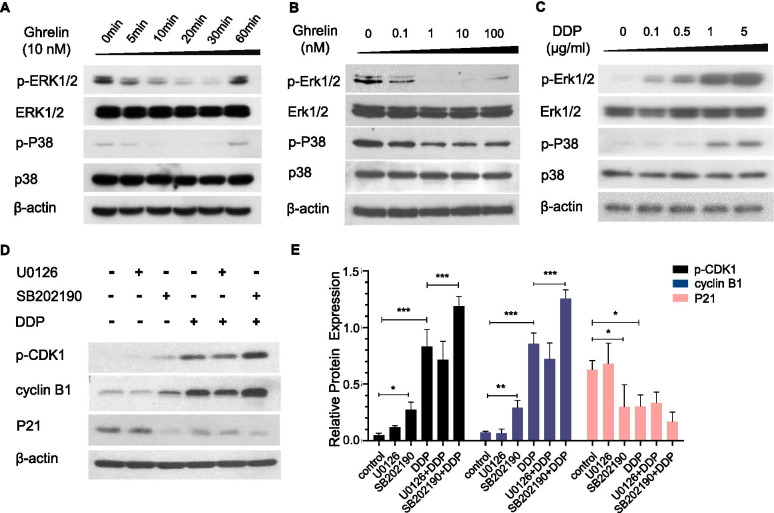
Signaling pathways involved in the treatment with ghrelin and cisplatin. **A-B** Western blotting analysis of the expression of p-ERK1/2 and p-p38 under treatment with ghrelin in time-dependent (10 nM) (**A**) and dose-dependent (**B**) manners for 20 min. **C** Western blotting analysis of the expression of p-ERK1/2 and p-p38 with the treatment of cisplatin for 12 h in dose-dependent manners. **D** Western blotting analysis of the expression of p-CDK1, cyclin B1, and p21 in HO-8910 PM treated with MAPK inhibitors (U0126 or SB202190, 5 μM, respectively), cisplatin and the combination of inhibitors with cisplatin for 12 h. Data are shown as the mean ± SD from three independent experiments. *p < 0.05

To understand how ghrelin and cisplatin regulated proteins involved with cyclin B1 and p-CDK1, we investigated the signaling pathway in HO-8910 PM after ghrelin and cisplatin treatment. We found that ghrelin inhibited p-ERK1/2 and p-p38 in time and dose-dependent manners, which reached an optimal effect at a dosage of 10 nM at 20 min after ghrelin treatment (Fig. 5A-B). However, it did not alter p-AKT expression (data not shown). Unexpectedly, cisplatin treatment promoted p-ERK1/2 and p-p38 expression in dose-dependent manners (Fig. 5C). We hypothesized that the increased expression of p-ERK1/2 and p-p38 was a negative feedback action in response to cisplatin treatment. We further investigated the effect of cisplatin on cell cycle protein regulation followed by U0126 and SB202190. We found that U0126 treatment slightly increased p-CDK1 expression but did not alter cyclin B1 or P21 expression (Fig. 5D-E). SB202190 treatment significantly increased the expression of p-CDK1 and cyclin B1 but decreased the expression of P21. Combined with cisplatin, SB202190 treatment dramatically promoted the expression of p-CDK1 and cyclin B1, and slightly decreased P21 expression (Fig. 5D-E). However, U0126 treatment did not alter the expression of these proteins induced by cisplatin. These data suggested that p38 may be the primary factor mediating ghrelin promoted cisplatin sensitivity.

### Inhibitors of p38 enhanced sensitivity of HO-8910 PM to cisplatin

**Fig. 6:**
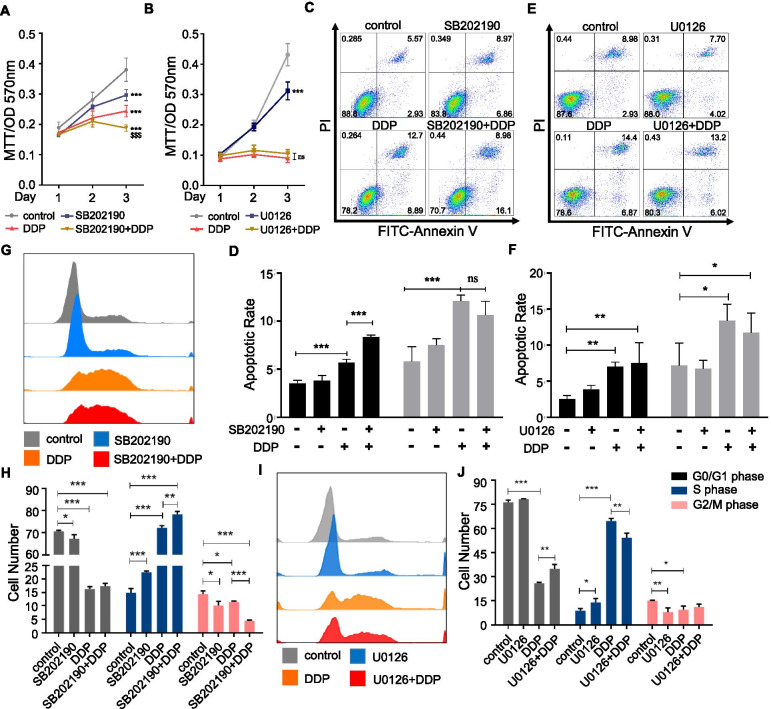
Inhibitors of p38 enhanced sensitivity of HO-8910 PM to cisplatin. **A**-**B** MTT assay of HO-8910 PM under U0126 (5 μM) or SB202190 (5 μM) treatment in the presence or absence of cisplatin (1 μg/ml). **C**-**F** Alterations of the apoptosis measured in HO-8910 PM after treatment with U0126 (**C**-**D**) or SB202190 (**E**-**F**), cisplatin and the combination of inhibitors with cisplatin. **G**-**J** The cell cycle distribution of HO-8910 PM after treatment with U0126 (**G**-**H**) or SB202190 (**I**-**J**), cisplatin and the combination of inhibitors with cisplatin. Data are shown as the mean ± SD from three independent experiments. * *p* < 0.05, ** *p* < 0.01, ***p < 0.001 versus control group. $$$ *p* < 0.001 versus DDP group

We subsequently investigated whether the MAPK signaling pathway was involved in cell phenotypes elicited by cisplatin treatment. We found that both U0126 and SB202190 inhibited cell viability with or without cisplatin (Fig. 6A-B). In the cell apoptosis assay, SB202190 treatment slightly increased early apoptosis when treated alone, and significantly increased early apoptosis when treated with cisplatin (*p* < 0.001); however, it did not alter late apoptosis (Fig. 6C-D). U0126 treatment increased early apoptosis too, but it did not alter early apoptosis induced by cisplatin nor the late apoptosis (Fig. 6E-F).

Moreover, we investigated the effects of ERK1/2 and p38 on the cell cycle under cisplatin treatment. Inhibition of ERK1/2 slightly increased cell populations in S phase and attenuated cell populations in G2/M phase (Fig. 6I-J); inhibition of p38, like U0126 treatment, significantly increased S phase progress but suppressed G0/G1 phase and G2/M phase progress (Fig. 6G-H). However, under the treatment of cisplatin, SB202190 exerted an opposite effect on cell cycle regulation as compared with U0126. Inhibition of ERK1/2 reversed the regulation effect of cisplatin on G0/G1 phase inhibition and S phase arrest (Fig. 6I-J). However, inhibition of p38 enhanced the regulatory effect of cisplatin on S phase arrest and G2/M phase inhibition (Fig. 6G-H). These data suggested that the p38 signaling pathway was a key factor mediating ghrelin induced S phase cell cycle arrest.

### Ghrelin specifically inhibited HO-8910 PM

**Fig. 7:**
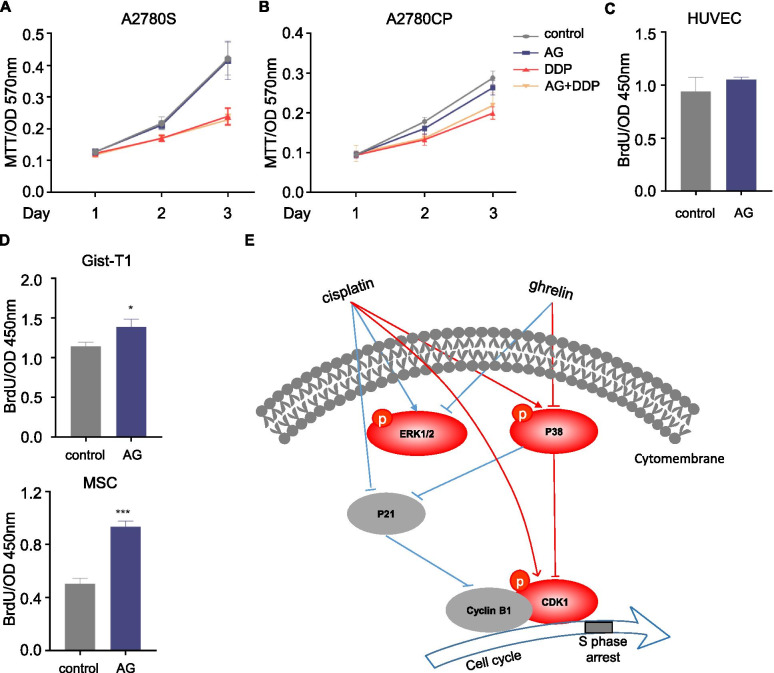
Ghrelin specifically inhibited HO-8910 PM. **A-B** MTT assay of A2780S (**A**) and A2780CP (**B**) under ghrelin treatment (10 nM) in the presence or absence of cisplatin (1 μg/ml). **C**-**D** The BrdU incorporation by HUVEC (**C**), Gist-T1 and iPSC-MSC (**D**) under the treatment of ghrelin. **E** A schematic showing the effects of ghrelin sensitizing HO-8910 PM to cisplatin. * *p* < 0.05, ***p < 0.001

To determine whether ghrelin is a broad-spectrum factor to suppress cancers, we detected the effects of ghrelin on cell proliferation with or without cisplatin in a series of cell lines, including epithelial cells, mesenchymal cells, and endothelial cells. Unexpectedly, it did not alter cell proliferation in A2780S or A2789CP (Fig. 7B), another two ovarian cancer cell lines. In human umbilical vein endothelial cells (HUVEC), ghrelin did not affect cell proliferation either (Fig. 7C). In mesenchymal cells, ghrelin treatment increased cell proliferation in both iPSC-MSC and GIST-T1 (Fig. 7D). These data indicated that ghrelin specifically suppressed some ovarian cancer cells.

## Discussion

Ovarian cancer is the second most common type of lethal gynecologic malignancy of the female population all throughout the world [21]. In China, both the age-standardized incidence rate (ASIR) and age-standardized mortality rate (ASMR) showed an ascending trend during the period from 2000 to 2013, with average annual percent change (AAPC) values of 1.4 and 4.5%, respectively [22]. The lack of obvious symptoms and effective screening leads to the late stage diagnosis. Moreover, the development of drug resistance, like resistance to cisplatin, further decreases the survival.

Ghrelin is involved in a series of cancer progression, but its roles in ovarian cancer are largely unknown. In this article, we studied its effects on the phenotypes and chemoresistance of ovarian cancer cell lines. We found that, in HO-8910 PM, treatment by ghrelin inhibited cell proliferation, cell viability and clone formation, which were in line with previous reports [23, 24].

To determine the effects of ghrelin on the chemosensitivity of ovarian cancer to cisplatin, HO-8910 PM was exposed to ghrelin combined with cisplatin. As expected, treatment with ghrelin enhanced the suppressive effect of cisplatin on cell proliferation and cell viability of HO-8910 PM. The data were supported by the animal studies. We found that, in the HO-8910 PM transplanted nude mice, tumor volume and tumor weight were both inhibited under the treatment with ghrelin and cisplatin, and were further inhibited when treatment with ghrelin was combined with cisplatin. The data suggested that ghrelin treatment enhanced the sensitivity of HO-8910 PM to cisplatin. Noted is that, though treatment of ghrelin enhanced the cytotoxicity of cisplatin to the tumor derived from HO-8910 PM, it did not enhance the side effects of cisplatin to the mice, because ghrelin treatment did not influence the body weight of recipient mice.

The mechanisms of acquired resistance can be broadly classified into three major groups that are related to (i) the tumor microenvironment; (ii) cancer stem cells; and (iii) the DNA repair pathways [4]. Among the many mechanisms of drug resistance described in vitro, increased repair of platinum DNA damage appears to be the most important factor [25]. Maintenance of genome stability relies on the precise orchestration of DNA replication, chromosome segregation, DNA repair and genome surveillance mechanisms, along with their integration with cell cycle progression and myriad other processes such as transcription and metabolism [26]. If NDA damage is not repaired, the consequence is a block of DNA synthesis and transcription and cell cycle progress [27].

To understand the possible mechanisms for inhibition of cell growth, the cell cycle and cell apoptosis were measured. Under the treatment of ghrelin, a dose-related significant increase of cells in S phase was noted, along with a significant decrease in the G2/M phase. However, no effects on cell apoptosis were observed. Following the treatment with cisplatin, ghrelin significantly lowered the percentages of G0/G1 and G2/M phases, but caused a dramatic increase of cells in S phase.

To further confirm the regulation of ghrelin and cisplatin on cell cycle, cell cycle associated proteins p-CDK1, cyclin B1 and P21 were analyzed. P21 was reported to degrade cyclin B1 [28], inhibit S/G2 phase transition, and cause S phase cell cycle arrest [29]. Unexpectedly, compared to the cisplatin group, an increased accumulation of cyclin B1 was observed in the group treated with ghrelin combined with cisplatin; correspondingly, the expression of p21 decreased. These findings seemed to contradict those of the cell cycle assay. To investigate the causes of S phase cell cycle arrest, we further determined the expression of p-CDK1, because dephosphorylation of CDK1 at Tyr15 and Thr14 is a critical regulatory step in activating CDK1 during progression into mitosis [30]. As expected, we observed a stronger accumulation of p-CDK1 in the group of cisplatin combined with ghrelin than in the group of cisplatin and ghrelin. These data suggested that treatment with ghrelin enhanced the sensitivity of HO-8910 PM to cisplatin via p-CDK1 mediated S phases cell cycle arrest.

Ghrelin regulates cell phenotypes via the MAPK signaling pathway. Therefore, we determined the effects of ERK1/2, p38 and AKT signaling pathway in chemosensitivity of HO-8910 PM to cisplatin. It is an interesting finding that the expression of p-ERK1/2 and p-p38 were inhibited under the treatment of ghrelin but elevated under the treatment of cisplatin. However, it was the inhibitor of p38, but not inhibitors of ERK1/2, that promoted the effects of cisplatin to upregulate the expression of p-CDK1 and cyclin B1, inhibit the expression of P21, and to suppress cell proliferation and cell cycle progression of HO-8910 PM.

To determine whether ghrelin is a broad-spectrum inhibitor in ovarian cancer, we examined the cell proliferation or cell viability in ovarian cancer cell lines A2780S and its cisplatin resistant subset A2780CP, HUVEC endothelial cells, and mesenchymal cells Gist-T1 and iPSC-MSCs under the treatment of ghrelin or cisplatin. Treatment with ghrelin promoted the cell proliferation of mesenchymal cells Gist-T1 and MSCs, but not A2780S, A2780CP and HUVEC. These data indicate that ghrelin enhances sensitivity to cisplatin in specific clinical types of ovarian cancer, but not all ovarian cancers. This may because that ovarian cancer encompasses complex subtypes differentiated by cell/site of origin, pathological grade, risk factors, prognosis, and treatment [31]. Typically, more aggressive than non-epithelial malignancies, epithelial cancers are most common among women of all racial/ethnic groups, accounting for 90% of all cases. Epithelial cancers are classified by tumor cell histology as serous (52%), endometrioid (10%), mucinous (6%), or clear cell (6%), with one-quarter being rarer subtypes or unspecified. Epithelial malignancies are further grouped as type I or type II based on clinical pathologic factors.

## Conclusion

In summary, we reported that treatment with ghrelin specifically promoted cisplatin sensitivity by inducing phosphorylation of CDK1 at Tyr15 and Thr14 via inhibiting the phosphorylation of p38, and subsequently promoting S phase cell cycle arrest in the HO-8910 PM ovarian cancer cell line. These data suggest that the level of ghrelin and the tumor subtypes in some patients with ovarian cancer may influence the chemosensitivity and outcome. Furthermore, p38 is a potential target to inhibit platinum-resistance.

## Data Availability

The datasets used and/or analyzed during the current study are available from the corresponding author on reasonable request.
